# SNP-Associated Substitutions of Amino Acid Residues in the dNTP Selection Subdomain Decrease Polβ Polymerase Activity

**DOI:** 10.3390/biom14050547

**Published:** 2024-05-02

**Authors:** Olga A. Kladova, Timofey E. Tyugashev, Aleksandr A. Miroshnikov, Daria S. Novopashina, Nikita A. Kuznetsov, Aleksandra A. Kuznetsova

**Affiliations:** 1 Institute of Chemical Biology and Fundamental Medicine, Siberian Branch of Russian Academy of Sciences, 630090 Novosibirsk, Russia; tyugashev@niboch.nsc.ru (T.E.T.); danov@niboch.nsc.ru (D.S.N.); nikita.kuznetsov@niboch.nsc.ru (N.A.K.); 2 Department of Natural Sciences, Novosibirsk State University, 630090 Novosibirsk, Russia; a.miroshnikov@g.nsu.ru

**Keywords:** DNA repair, DNA polymerase beta, single-nucleotide polymorphism, enzymatic activity

## Abstract

In the cell, DNA polymerase β (Polβ) is involved in many processes aimed at maintaining genome stability and is considered the main repair DNA polymerase participating in base excision repair (BER). Polβ can fill DNA gaps formed by other DNA repair enzymes. Single-nucleotide polymorphisms (SNPs) in the *POLB* gene can affect the enzymatic properties of the resulting protein, owing to possible amino acid substitutions. For many SNP-associated Polβ variants, an association with cancer, owing to changes in polymerase activity and fidelity, has been shown. In this work, kinetic analyses and molecular dynamics simulations were used to examine the activity of naturally occurring polymorphic variants G274R, G290C, and R333W. Previously, the amino acid substitutions at these positions have been found in various types of tumors, implying a specific role of Gly-274, Gly-290, and Arg-333 in Polβ functioning. All three polymorphic variants had reduced polymerase activity. Two substitutions—G274R and R333W—led to the almost complete disappearance of gap-filling and primer elongation activities, a decrease in the deoxynucleotide triphosphate–binding ability, and a lower polymerization constant, due to alterations of local contacts near the replaced amino acid residues. Thus, variants G274R, G290C, and R333W may be implicated in an elevated level of unrepaired DNA damage.

## 1. Introduction

Effects of various environmental factors acting on the cell constantly lead to damage to genetic information encoded in DNA [1]. Such lesions can cause mutations in the genome, which means defects in the integrity of the genetic information [2,3,4,5]. To maintain genome stability, living organisms have several DNA repair systems that preserve their original sequence [6,7]. One such system is base excision repair (BER), which involves the action of many enzymes [8]. The first to find and recognize a base lesion are DNA glycosylases, which remove the damaged base [9,10,11]. The resulting apurinic/apyrimidinic (AP) site can then be hydrolyzed by AP endonuclease 1 [12,13]. The next enzyme acting in this pathway is DNA polymerase β (Polβ) [14]. The latter is considered the main repair DNA polymerase whose main function is to fill the gaps in DNA (formed after the action of AP endonuclease 1) with complementary deoxynucleotide triphosphates (dNTPs) via the short-patch BER pathway [15]. Under certain conditions, Polβ can continue the synthesis by generating longer DNA fragments with strand displacement via the long-patch pathway [16,17].

Polβ is a small protein with a mass of 39 kDa. The enzyme has two structural domains: dRP-lyase and nucleotidyl transferase [15,18,19]. The nucleotidyl transferase domain can be functionally divided into several subdomains: the DNA-binding subdomain, the nucleotidyl transferase subdomain, and the subdomain of dNTP selection. One of the most important functions of Polβ is to fill a gap in DNA with a complementary nucleotide monophosphate (dNMP). The correct choice of complementary dNTP is necessary to prevent mutations. The interactions of the dNTP selection domain (amino acid residues [aa] 269–335) with incoming dNTP are critical for the selection of a complementary dNTP to incorporate.

Amino acid residues of Polβ form many contacts with substrates (DNA and dNTP); disruption of the extensive network of the interactions because of substitutions of amino acid residues in the enzyme can result in changes in the activity of the enzyme. Such amino acid substitutions can arise due to the occurrence of natural single-nucleotide polymorphisms (SNPs) in the genome [20]. It is known that the genes of DNA repair enzymes contain many SNPs, some of which considerably affect their function [21,22]. Most SNPs found in the *POLΒ* gene are localized to introns or do not lead to a change in the functional class of an affected amino acid residue. Analysis of various polymorphisms has made it possible to detect various SNPs that alter the class of the amino acid residue in the enzyme (missense mutations) and can therefore affect its function [23]. In the dNTP selection subdomain, there are amino acid substitutions (described in databases) caused by SNPs found in patients with various tumors (Table 1).

For some SNPs located in the dNTP selection subdomain, the effect on enzymatic properties of Polβ is known. For example, variant Glu288Lys has been reported to have lower fidelity of dATP incorporation into single-nucleotide (1-nt) gap-containing DNA [24]. At the same time, dissociation constants *K*_d_, polymerase reaction rate constant *k*_obs_, and rate of product release *k*_ss_ are similar between the Polβ variant and wild type (WT) Polβ. The Lys289Met variant more frequently attaches dCTP opposite cytidine in DNA, thereby showing poor discrimination of dNTPs during the transferase reaction [25,26]. It has been demonstrated that the rate constant of dNTP incorporation into 1-nt gap-containing DNA in this variant is considerably lower than that of WT Polβ. Meanwhile, the dissociation constant *K*_d_ is virtually the same. The polymorphic variant containing the Glu295Lys substitution does not possess polymerase activity, thereby leading to the emergence of unfilled gaps in DNA. This mutant of Polβ has been detected in patients with gastric carcinoma [27].

**Figure 1:**
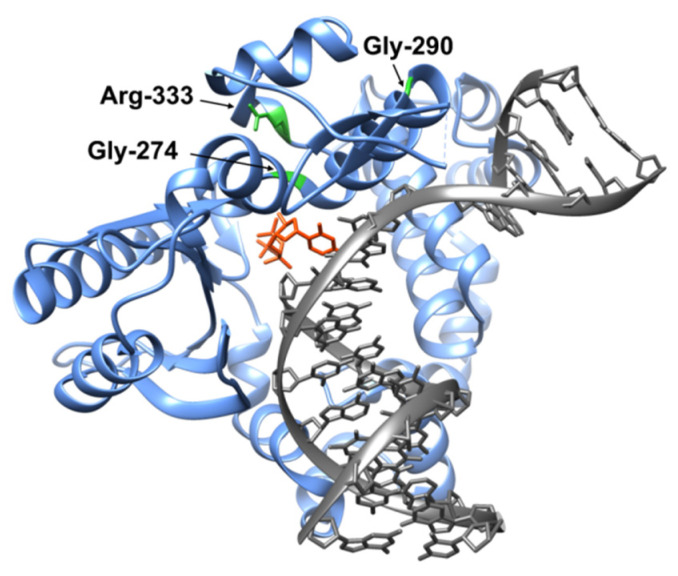
The ternary complex of Polβ with DNA containing a 1-nt gap and a modified dCTP analog (orange). Residues Gly-274, Gly-290, and Arg-333 are highlighted in green in the structure. DNA is gray, and the enzyme is blue. Protein Data Bank (PDB) ID: 5UGP.

We recently analyzed known SNPs in the *POLB* gene that lead to a change in the class of an amino acid residue and can therefore disrupt the function of the amino acid residue [23]. As a result of the analysis, 196 substitutions of amino acid residues were identified, among which 33 substitutions were located in the dNTP selection subdomain. They were ranked according to the possible negative impact on the functioning of the Polβ enzyme. In the dNTP selection subdomain, several substitutions were found with a high risk of impairment of the enzyme’s action, including substitutions G274R, G290C, E316K, P330L, R333Q, and R333W, located at the C terminus of the enzyme (Figure 1). The role of some of these amino acid residues has already been discussed in the literature [28,29]. Unstudied SNP variants G274R, G290C, and R333W were selected for further analysis.

Gly-274 is a conserved residue located between DNA helices M and N in a protein region undergoing considerable conformational rearrangements [30]. The substitution of Gly-274 with Val has been identified in melanoma patients (databases COSMIC and cBioportal). Gly-290 is located at a distance from the active center of the enzyme; in the binary complex with DNA and in the ternary complex with DNA and dNTP, this residue becomes closer to DNA [15,18,31]. Arg-333 is located in a poorly structured region of the C-terminal tail [18]. Despite the distal localization from the active center, it is known that some functional mutations of the amino acid residue at position 333 induce almost complete inactivation of the enzyme [29]. The current work presents a study about the enzymatic properties of SNP-associated Polβ variants G274R, G290C, and R333W, and the influence of these amino acid substitutions on local contacts in the enzyme.

## 2. Materials and Methods

### 2.1. Protein Purification

A pET28-c plasmid encoding human WT Polβ (as the *POLB* gene) or its variant G274R, G290C, or R333W (as *POLB* carrying an SNP) was transfected into *Escherichia coli* Rosetta 2 cells. Proteins’ expression was induced by the addition of 0.1 mM isopropyl β-D-thiogalactopyranoside (IPTG) after optical density at 600 nm (OD_600_) reached 0.5–0.6 before harvesting of the cells via centrifugation. Cell pellets were resuspended in a buffer (20 mM HEPES-KOH, 40 mM NaCl, pH 7.8) and lysed via a French press. The lysate was centrifuged at 40,000× *g* for 40 min. For enzymes purification, two-stage chromatography on a Q-Sepharose column and Ni-NTA resin was performed. The collected protein fraction was dialyzed overnight against a buffer composed of, 20 mM HEPES-KOH pH 7.8, 150 mM NaCl and 20% glycerol. The pure protein fraction was supplemented with 50% glycerol and stored at −20 °C. For more details, refer to [32].

### 2.2. DNA Substrates

Sequences of the oligodeoxyribonucleotides (containing a gap with 2′-deoxyribose phosphate) used in the work are given in Table 2. The 36-bp DNA substrates used in DNA-binding, strand displacement synthesis, and gap-filling assays were labeled with FAM at the 5′ end of the primer strand and obtained by mixing equimolar amounts of three DNA strands: FAM_Pol19, Pol_36_N, and Pol16. The DNA substrate subjected to the conformational dynamics analysis and containing a 2-aminopurine (2-aPu) residue was prepared by mixing equimolar amounts of Pol16, Pol19, and Pol36_T_Ã. The DNA substrates were annealed for 5 min at 93 °C and allowed to cool down to room temperature.

### 2.3. Circular Dichroism Spectroscopy (CD)

CD spectra were recorded on a Jasco J-600 spectropolarimeter (Jasco, Tokyo, Japan). The enzyme solutions were prepared in a buffer composed of 50 mM Tris-HCl, pH 7.5, 50 mM KCl, 1.0 mM EDTA, and 5.0 mM MgCl_2_, and were placed in quartz cuvettes with a light path length of 0.1 mm. Spectra with a bandwidth of 1.0 nm and a wavelength ranging from 190 to 260 nm were acquired. Automatic averaging was done when the measurements were taken. An online tool for choosing and modeling protein CD spectra was used to describe the spectra [33,34,35].

### 2.4. Analysis of the Melting Point of the Enzymes

The 50 µM samples of enzymes were prepared in a buffer (50 mM Tris-HCl pH 7.5, 50 mM KCl, 1.0 mM EDTA, 5.0 mM MgCl_2_) and supplemented with 5× ProteOrange dye (Lumiprobe, Moscow, Russia). Melting points were measured by means of a Quant Studio 5 real-time PCR system (Applied Biosystems, Waltham, MA, USA) in PCR tubes using the thermal shift assay. The temperature was constantly raised in steps of 0.028 °C, from 25.1 to 99.9 °C. The fluorescence intensity of the ProteOrange dye was recorded via excitation at 470 nm and emission at 558 nm. Each melting point value was calculated using the Boltzmann sigmoid curve equation:

F = F_u_ + (F_b_ − F_u_)/{1 + exp(T_m_ − x/slope)}, (1)

where F is ProteOrange fluorescence emission, x is temperature, F_u_ is baseline fluorescence at low temperature, F_b_ is maximum fluorescence at high temperature, the slope describes the steepness of the curve, and T_m_ is the melting point of the protein.

### 2.5. DNA-Binding Analysis

The DNA–Polβ complexes were separated in a non-denaturing 10% PAAG (the ratio of acrylamide to *N*,*N*′-methylenebisacrylamide was 75:1) at 4 °C. The DNA-binding reaction samples were prepared in a buffer composed of 50 mM Tris-HCl pH 7.5, 50 mM KCl, 1 mM Na_2_EDTA, 5 mM MgCl_2_, 1 mM DTT, and 7% glycerol. The recombinant enzymes were serially diluted; for the Polβ G274R polymorphic variant, the concentration range was from 86 nM to 11 μM; for the G290C variant, the concentration range was 0.16 to 20 μM; and for the R333W variant, the concentration range was 0.9 to 115 μM. The concentration of FAM-labeled substrate Gap_N was 50 nM. To determine the dissociation constant, the resultant gel was visualized in a VersaDoc gel-documenting system (Bio-Rad Laboratories, Hercules, CA, USA). The results were processed using the Gel-Pro Analyzer 4 software (Media Cybernetics, Rockville, MD, USA). The dissociation constant *K*_d_ for each enzyme–DNA complex was computed in the OriginPro 8 software via the equation:

Formed complex (%) = F_u_ + (F_b_ − F_u_)/{1 + (*K*_d_/[Polβ])h}, (2)

where h is the Hill coefficient, F_u_ is the correction for background illumination, and F_b_ is the maximum intensity of the complex.

### 2.6. Analysis of Polβ Variants’ Polymerase Activity

The DNA elongation assay was performed in a buffer consisting of 50 mM Tris-HCl pH = 7.5, 50 mM KCl, 1 mM Na_2_EDTA, 5 mM MgCl_2_, 1 mM DTT, and 7% glycerol at 37 °C. The 5 μL reaction samples contained 0.5 µM Gap_T substrate, 5 µM dNTP mix, and 0.5 µM enzyme. The reaction time was 1 min, and the reaction was stopped by mixing with 5 μL of a stop solution (7.5 M urea, 25 mM EDTA, 0.1% oxylene cyanol, and 0.1% of bromophenol blue). The separation of the reaction products was performed by denaturing 20% PAAG. 

To determine the gap-filling activity of the G290C variant, the reaction mixture containing 0.5 µM Gap_N substrate, 5 µM complementary dNTP, and 0.5 µM enzyme was used. The reaction was stopped by mixing with 5 μL of a stop solution (7.5 M urea, 25 mM EDTA, 0.1% xylene cyanol, and 0.1% bromophenol blue) after 20, 40, 60, 90, or 120 s. The prepared samples were applied to a denaturing 15% PAAG.

All resulting gels were visualized in the VersaDoc gel-documenting system (Bio-Rad Laboratories, Hercules, CA, USA). The data were processed using the Gel-Pro Analyzer 4 software (Media Cybernetics, Rockville, MD, USA) and the degree of substrate conversion was determined by means of the ratio of the peak areas of the product to the sum of the peak areas of the product and the peak of the initial substrate. A relevant characteristic of the polymerase activity of Polβ is the observed rate constant of the reaction of incorporation of various dNTPs into a synthesized DNA strand, *k*_obs_. The final calculation of the observed reaction rate constant was carried out in the OriginPro 8 software via plotting of the dependence of the product accumulation on reaction time. The obtained data were fitted to the equation:

[Product] = [S] × {1 − exp(−*k*_obs_ × t)}, (3)

where [S] is the initial concentration of the substrate, t is reaction time, and *k*_obs_ is the observed rate constant of the chemical reaction.

### 2.7. Registration of Conformational Changes in the DNA Substrate by the Stopped-Flow Method and Determination of Polymerization Reaction Rate Constant k_pol_ and Observed Constant K_d,app(dATP)_ for the G290C Variant

Conformational changes in the DNA substrate containing a gap and 2-aPu residue were recorded at a fluorescence excitation wavelength of 310 nm. Registration of conformational changes in the substrate DNA was carried out on an SX.20MV stopped-flow spectrophotometer (Applied Photophysics, Leatherhead, UK).

A comparison of the effects of variants G274R, G290C, and R333W on the conformational dynamics of DNA was carried out at 37 °C. The concentration of the enzymes was 1.0 μM, Gap_TÃ DNA substrate concentration was 0.5 μM, and dATP concentration was 100 μM.

To determine the influence of the substitution of the amino acid residue in variant G290C on the catalytic step and the step of enzyme binding to dATP (formation of a ternary complex), the experiment was conducted by varying the concentration of dATP. Concentrations of the enzyme and DNA substrate were 1.0 and 0.5 μM, respectively; varying concentrations of dATP were 50, 100, 150, 200, and 400 μM. The reaction was carried out at 37 °C.

To determine the polymerization reaction rate constant *k*_pol_ and observed constant *K*_d,app(dATP)_ of dissociation of dATP from the enzyme–DNA complex, the region of the fluorescence curves corresponding to the slow stage of the decrease in fluorescence intensity of 2-aPu was fitted to the following equation [36]:

F = F_0_ + F_1_ × exp(−*k*_obs_ × t), (4)

where F is the observed 2-aPu fluorescence intensity signal, F_0_ is background fluorescence, F_1_ is a fluorescence parameter, and *k*_obs_ is the observed rate constant. From the obtained values of the observed rate constants, a dependence on the concentration of dATP was constructed. The resulting dependence was fitted to Equation (4), which enabled us to obtain parameters *k*_pol_ and *K*_d,app(dATP)_:

*k*_obs_ = *k*_pol_[dATP]/(*K*_d,app(dATP)_ + [dATP]), (5)

where *k*_obs_ is the observed rate constant of the reaction, *k*_pol_ is the rate constant of the polymerization reaction, and *K*_d,app(dATP)_ is the dissociation constant of the enzyme–DNA–dATP complex.

### 2.8. Determination of Polymerization Reaction Rate Constant k_pol_ and Observed Constant K_d,app(dATP)_ for Variants G274R and R333W

To determine the influence of substitutions of amino acid residues in variants G274R and R333W on the catalytic step and the step of enzyme binding to dATP (formation of a ternary complex), the assay of separation of the reaction product in 15% PAAG was used. The experiments were conducted by varying the concentration of dATP. Concentrations of the G274R and R333W variants and DNA substrate were 1.0 and 0.5 μM, respectively. The varying concentrations of dATP were 25, 75, 100, 200, and 400 μM for the G274R variant and 25, 50, 75, 150, 250, and 750 μM for the R333W variant. The reaction was allowed to proceed at 37 °C. All resulting gels were visualized in the VersaDoc gel-documenting system (Bio-Rad Laboratories, Hercules, CA, USA). Observed rate constants *k*_obs_ were calculated by means of Equation (3). The polymerization reaction rate constant *k*_pol_ and observed constant *K*_d,app(dATP)_ of dissociation of dATP from the enzyme–DNA complex was calculated using Equation (5) from the resulting dependency of *k*_obs_ on dATP concentration.

### 2.9. Molecular Dynamic Simulations (MD)

Human Polβ apoenzyme structure was modeled by means of a crystal structure of rat Polβ [37]. Models of the Polβ–DNA open binary complex and Polβ–DNA–dNTP ternary closed complex were based on crystal structures of human Polβ complexes [15,38]. The DNA was edited to match experimental sequences, and the protein and the DNA primer were parametrized with the AMBER 14SB-OL15 force field set [39,40,41]. Homology and unstructured-region modeling was performed using Modeller [42]. Protein protonation states were assigned by PDB2PQR server with PROPKA [43,44]. Simulations were run with the help of the GROMACS 2022 MD package. A simulation box was set up with TIP3P water and 50 mM KCl JC ions [45,46]. Octahedral dummy model treatment was chosen for active-site magnesium ions [47]. RESP charges for nucleoside triphosphates were assigned using R.E.D. Server via an established approach [48,49]. Force field parameters were converted with ACPYPE [50]. The cutoff of nonbonded interactions was set to 0.8 nm, and long-range electrostatic interactions were treated via the PME method [51]. Bonds of hydrogen atoms were constrained using LINCS [52]. Flat-bottom potential restraints were applied to hydrogen-bonded heavy atoms of terminal base pairs in the truncated DNA primers. Steepest descent energy minimization was followed by 1 ns NVT and NPT equilibrations with heavy atom restraints. Unrestrained MD simulations were run in triplicate for 0.5 and 2 μs for the N-terminal fragment using a V-rescale thermostat and C-rescale barostat [53,54]. Resultant trajectories were processed with the integrated GROMACS toolset. PCA calculations were performed and plotted in NMWiz.

## 3. Results

### 3.1. Effects of the Substitutions on Protein Structure and Melting Temperature

**Figure 2:**
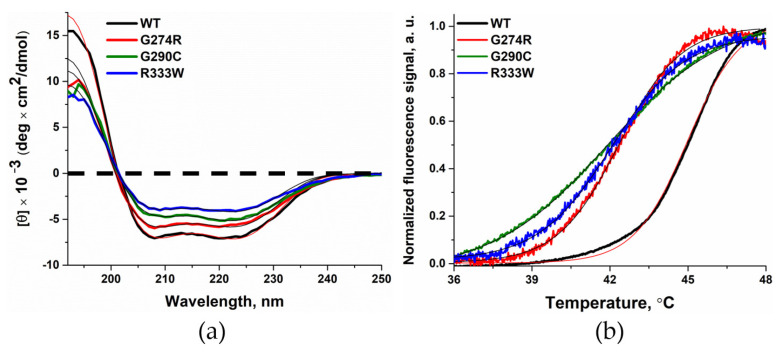
The influence of substitutions G274R (red), G290C (green), and R333W (blue) on Polβ secondary structure (**a**) and thermal stability (**b**). Data on WT Polβ, G274R, G290C, R333W are shown in black, red, green and blue respectively. Fitted lines for G274R, G290C, and R333W are indicated as thin black lines; fitted lines for WT Polβ are indicated as thin red lines.

To test the impact of the selected amino acid substitutions on protein structure, experiments were conducted to obtain circular dichroism (CD) spectra (Figure 2a). The spectra were fitted using the https://bestsel.elte.hu/ resource (accessed on 16 February 2023). The shape of the spectra obtained for variants G274R, G290C, and R333W had little difference from the spectrum obtained for WT Polβ. The calculated amount of α-helices contained in WT Polβ was almost two times greater than that in the studied variants of the enzyme (Table 2), possibly indicating greater mobility of variants G274R, G290C, and R333W.

To clarify the effects of the substitutions on the physical properties of Polβ, the melting temperature of the variants was measured by the thermal shift method. The enzyme samples were mixed with the intercalating dye ProteOrange and heated from 25.1 to 99.9 °C. The melting curves of WT Polβ and its variants had similar shapes (Figure 2b). The melting temperature of variants G274R, G290C, and R333W, as calculated via fitting of the melting curves to Equation (1), was slightly lower than that of WT Polβ (Table 3). Previously, for mutants of Polβ containing various substitutions of amino acid residues, it has been reported that such substitutions also do not lead to changes in the overall structure of the enzyme.

**Figure 3:**
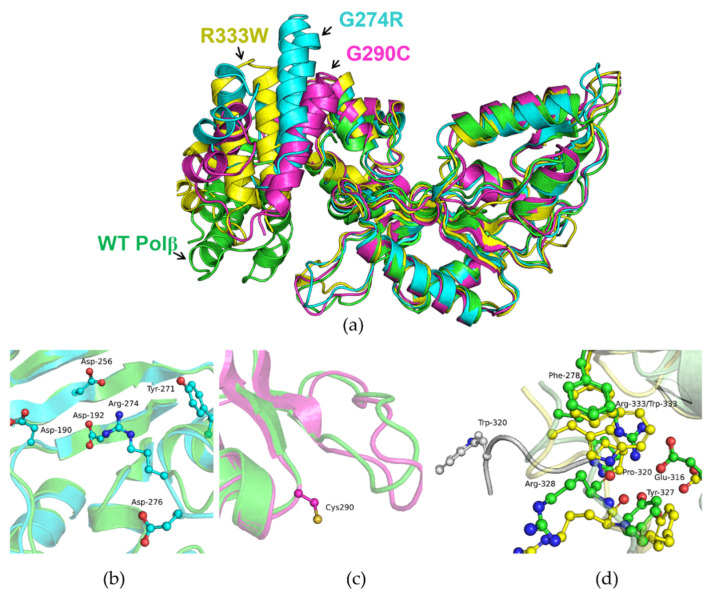
(**a**) An overlay of apoenzyme structures of WT Polβ and variants G274R, G290C, and R333W as a result of MD simulations. WT Polβ: green, G274R: blue, G290C: magenta, R333W: yellow. Close-ups of the microenvironment of amino acid residues Arg-274 (**b**), Cys-290 (**c**), Trp-333 (**d**). The variants of Polβ manifested similar MD and little difference from the WT enzyme.

For more detailed analysis, molecular dynamics (MD) simulations of the Polβ apoenzyme and of the polymorphic variants were performed next. Polβ is a protein of which considerable conformational mobility in the apoenzyme has been documented. The variants in question—G274R, G290C, and R333W—were found to have similar mobility in the MD simulations, and no considerable differences from WT Polβ were detectable (Figure 3a).

Examination of the MD simulation data on the immediate environment of the analyzed amino acid residues showed that the G290C polymorphic variant is the least different from the WT in this regard (Figure 3c). The WT Polβ apoenzyme and variant G290C retain a more open conformation. Cys-290 does not engage in any new contacts in contrast to WT Polβ.

For the G274R variant, it was revealed that the side chain of Arg-274 is mobile and can form hydrogen bonds and salt bridges with many catalytically important amino acid residues (Figure 3b) such as Asp-190, Asp-192, and Asp-256, coordinating the magnesium ion necessary for the transferase reaction.

Two stable structures were obtained for the R333W polymorphic variant. In the first one, which existed most of the time (Figure 3d, yellow), the introduction of the larger residue (Trp-333) caused a restructuring of the network of hydrogen bonding contacts. In this case, residue Trp-333 is in a stacking interaction with Pro-320 and Phe-278. The overall structure of the dNTP selection subdomain is preserved. The second possible consequence of the introduction of the R333W substitution may be the unfolding of the C-terminal region (Figure 3d, gray). Such protein folding may affect the activity of the enzyme.

Thus, the obtained data, together with the results of the thermal shift assay and analysis of the CD spectra, suggested that the introduction of substitution G274R, G290C, or R333W into Polβ does not lead to considerable structural changes, at least in the apoenzyme. Nonetheless, the G274R and R333W substitutions changed the natural local network of contacts near the respective amino acid residues, which may affect further enzymatic stages.

### 3.2. Determining the Effect on the Formation of the Complex with DNA

**Figure 4:**
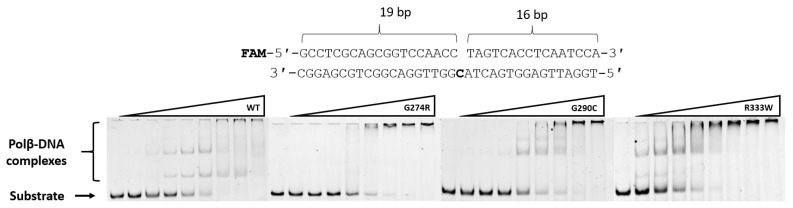
DNA-binding efficiency of WT Polβ and of polymorphic variants G274R, G290C, and R333W toward substrate Gap_C. Compared to the WT, the Polβ variants showed less effective complex formation with this DNA substrate. For the G274R variant, a clear-cut band related to the complex of the enzyme with DNA was less intense than for variants G290C and R333W. WT Polβ concentration: 22 nM to 2.63 μM, G274R concentration: 86 nM to 11 μM, G290C concentration: 0.16 μM to 20 μM, R333W concentration: 0.9 μM to 115 μM. The concentration of FAM-labeled DNA was 50 nM. Original figures can be found in Supplementary Materials.

To determine the effect of these amino acid substitutions on the stage of formation the binary complex with DNA, the electrophoretic mobility shift assay in a non-denaturing gel was used. DNA substrates contained a 1-nt gap with a different nucleotide placed in the opposite strand (Gap_A, Gap_T, Gap_G, and Gap_C) and were FAM-labeled on the elongated strand. The different complementary DNA strands, which allowed the placement of different nucleotides opposite the 1-nt gap, were employed to check the influence of various bases on the efficiency of binary enzyme–DNA complex formation. The structure of the DNA substrate is presented in Figure 4 and in the Materials and Methods section. For variants G274R, G290C, and R333W, several complexes (with DNA) having different mobility were detectable. The formation of such DNA complexes is also characteristic of WT Polβ [56].

**Figure 5:**
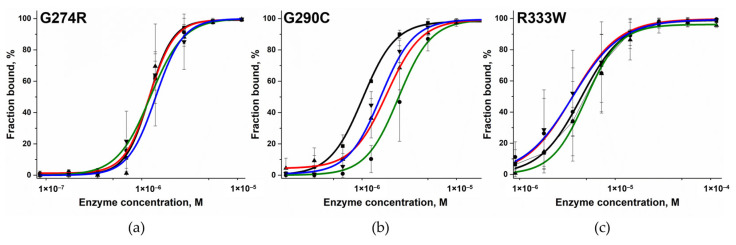
Complex formation efficiency of polymorphic variants G274R (**a**), G290C (**b**), and R333W (**c**) toward DNA substrates containing different nucleotides opposite the 1-nt gap [black: Gap_A (■), red: Gap_T (●), green: Gap_G (▲), and blue: Gap_C (▼)]. G274R concentration: 86 nM to 11 μM, G290C concentration: 0.16 μM to 20 μM, R333W concentration: 0.9 μM to 115 μM. The concentration of FAM-labeled DNA was 50 nM.

To characterize the impact of the substitutions G274R, G290C, and R333W on the efficiency of DNA-binding, the percentage ratio of free DNA to DNA in complex with the enzyme was calculated. From the dependence of the obtained ratio on the change in enzyme concentration in the reaction, the dissociation constant of the Polβ–DNA complex was calculated using Equation (2) (Figure 5). All the tested polymorphic variants had a weaker affinity for DNA than the WT enzyme did (Table 4). The obtained dissociation constant *K*_d_ values differed when DNA substrates containing different nucleotides opposite the 1-nt gap were analyzed. The effects on the formation of the complex with DNA were similar between substitutions G274R and G290C; a weakening of the affinity of the enzymes for DNA by approximately 3–4-fold was recorded on average. The largest increase in *K*_d_ was obtained for the R333W variant: by 10-fold on average, depending on the type of nucleotide located opposite the 1-nt gap in the DNA substrate.

**Figure 6:**
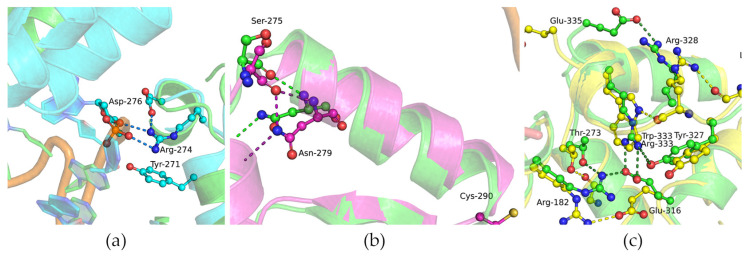
Overlays of snapshots of the dNTP selection subdomain in an open binary protein–DNA complex. The overlay of WT Polβ (green) and G274R (cyan) (**a**). The overlay of WT Polβ (green) and G290C (magenta) (**b**). The overlay of WT Polβ (green) and R333W (yellow) (**c**). Salt bridges between amino acid side chains and the sugar-phosphate backbone are shown as dashed lines.

For a more detailed understanding of possible rearrangements near the replaced amino acid residues during the formation of a complex with DNA, MD simulations of complexes of the Polβ variants with DNA were carried out next (Figure 6).

During the MD simulation analysis, it was revealed that the Arg-274 residue can form contact with the phosphate group of the nucleotide located opposite the gap and with the O5′ atom of the downstream nucleotide (Figure 6a).

In MD simulations of the complex of variant G290C with DNA, the changes introduced by the mutation were poorly distinguishable from the general background because the complexes have substantial mobility. The C-terminal region of the G290C polymorphic variant proved to be more mobile than that of WT Polβ. It was noted that the introduction of the cysteine residue at position 290 induces a redistribution of contacts in the binary complex: the amide group of Asn-279 forms hydrogen bonds with the backbone oxygen atom of Ser-275, while in the WT, bonds involving O(Leu-270) and O(Tyr-271) are more dominant (Figure 6b).

An even more pronounced redistribution of contacts occurred when Arg-333 was replaced by Trp (Figure 6c). This substitution impaired DNA-binding and increased the dissociation constant of the binary complex.

Taken together, these findings indicate an influence of substitutions G274R, G290C, and R333W on the stage of binding to the DNA substrate. Substitutions G274R and G290C led to a slight increase in the dissociation constant *K*_d_ and a slight redistribution of contacts near DNA-binding sites. In this context, variant R333W manifested the greatest difference from WT Polβ; the replacement of the R333 residue elevated *K*_d_ by 10-fold and induced a greater redistribution of contacts formed by Arg-333 in WT Polβ.

### 3.3. Determination of the Gap-Filling Efficiency of the Polβ Variants

**Figure 7:**
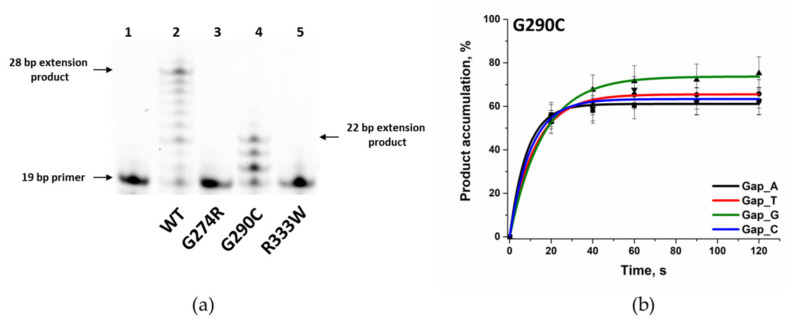
(**a**) A comparison of the effectiveness of strand replacement synthesis by Polβ variants G274R, G290C, and R333W. In lane 1 is 19-Nucleotide FAM-labeled DNA primer Gap_T, primer extension by WT Polβ is presented in lane 2, primer extension by the G274R variant is in lane 3, primer extension by the G290C variant is in lane 4, and primer extension by the R333W variant is in lane 5. WT Polβ generated products up to 30 nt in length within 1 min of the reaction by elongating the DNA primer by up to 11 nt. Variant G290C proved to be a less active polymerase compared to WT Polβ. The G290C variant extended the primer by 3 nt. Variants G274R and R333W did not elongate the DNA within the given reaction timeframe. The dNTP mix concentration was 10 μM, the enzymes’ and DNA concentrations were 0.5 μM, and the temperature was 37 °C. The reaction time was 1 min. (**b**) Single-nucleotide incorporation by the G290C variant. The dNTP concentration was 5 μM, the enzyme and DNA concentrations were 0.5 μM, and the temperature was 37 °C.

Many known amino acid substitutions generated by SNPs weaken polymerase activity of Polβ during the filling of a 1-nt gap and during extension of the primer strand [27,32,55,57]. To investigate the influence of amino acid substitutions G274R, G290C, and R333W on the polymerase activity of the enzyme, products of the enzymatic reaction were separated by polyacrylamide gel (PAAG) electrophoresis. The reaction was carried out by mixing a solution of a FAM-labeled DNA substrate containing a 1-nt gap, a complementary dNTP, and the enzyme. In the assay of the ability of the Polβ variants to elongate the DNA strand containing the 1-nt gap, it was shown that only the G290C variant can extend a DNA strand, whereas the attachment of new nucleotides occurred more slowly as compared to the WT enzyme (Figure 7a). For variants G274R and R333W under the chosen conditions (0.5 µM DNA, 0.5 µM enzyme, and 5 µM dNTP), the accumulation of the reaction product was undetectable, indicating a considerable degree of enzyme inactivation.

To evaluate the only ability of the G290C variant (gap-filling), an experiment was conducted in the presence of various triphosphates by means of four types of DNA substrates containing different nucleotides opposite the 1-nt gap (Figure 7b). Observed reaction rate constants *k*_obs_ were calculated using Equation (3) and were, on average, three times lower relative to WT Polβ (Table 5). Similarly to WT Polβ, the incorporation of dCMP by the variants into the 1-nt gap was the least efficient.

### 3.4. dNTP Binding and Incorporation

The detected change in the polymerase activity of the Polβ variants G274R, G290C, and R333W may be explained not only by the shift of affinity for 1-nt gap-containing DNA but also by a change in the ability to bind complementary dNTPs. For many polymorphic variants of Polβ, there is evidence of a decrease in observed dissociation constant *K*_d,app(dATP)_, as well as a decrease in polymerization constant *k*_pol_ (reflecting the rate of the chemical stage) [32,55]. 

It is known that when interacting with DNA and triphosphates, Polβ undergoes several stages of conformational alterations [36]. In this case, the changes in conformation occur not only in the enzyme but also in the DNA: it bends by ~90° [58]. To record such conformational changes, the stopped-flow method was used with a DNA substrate containing a 1-nt gap and a fluorescent base analog: 2-aminopurine (2-aPu). When the environment changes from hydrophobic to hydrophilic, a 2-aPu residue can change its fluorescence intensity [59,60,61]. To determine the binding parameters of variants G274R, G290C, and R333W here, various concentrations of complementary triphosphate were added to the reaction. 

**Figure 8:**
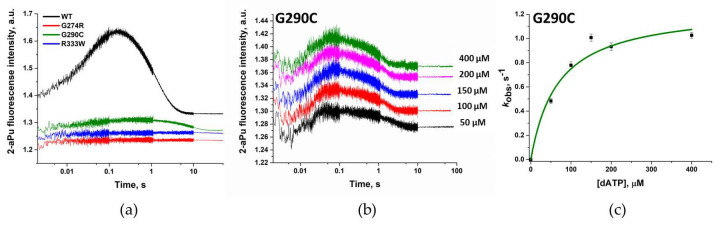
(**a**) A comparison of changes in the fluorescence intensity of a 2-aPu residue, which is part of a DNA substrate, during interaction with WT Polβ or variant G274R, G290C, or R333W. [Enzyme] = 1.0 µM, [Gap_TÃ] = 0.5 µM, [dATP] = 100 µM. (**b**) Changes in the fluorescence intensity of a 2-aPu residue incorporated into DNA during interaction with the G290C variant at various dATP concentrations. [Enzyme] = 1.0 µM, [Gap_TÃ] = 0.5 µM. (**c**) The dependence of calculated values of the observed reaction rate constants on the dATP for the polymorphic variant G290C.

The interaction of two almost inactive variants (G274R and R333W) with the DNA substrate did not alter the fluorescence intensity of the 2-aPu residue incorporated into the DNA (Figure 8a). The Trp residue has its own fluorescence too; conformational dynamics of many enzymes can be recorded when tryptophan residues are present in the protein globule [62]. WT Polβ contains one Trp (at position 325), whose change in intensity is insufficient to register the conformational dynamics of the enzyme. The introduction of an additional Trp (at position 333) into the protein globule also did not allow for the detection of changes in the fluorescence intensity of Trp residues.

The active variant G290C caused changes in the fluorescence intensity of the 2-aPu residue when interacting with the DNA substrate. The determined changes differed from those reported in the literature for WT Polβ (Figure 8a,b). The amplitude of the signal was lower compared with WT Polβ, whereas the phase of decline of 2-aPu fluorescence intensity observed after 0.2 s was within the same timeframe in comparison with WT Polβ. Earlier, it has been demonstrated that the decrease phase of fluorescence intensity of 2-aPu corresponds to the stage of accumulation of the reaction product [36]. To determine the binding parameters of polymorphic variant G290C toward dATP and to measure the polymerization constant, the part of the curve matching the decrease in fluorescence intensity of 2-aPu was fitted to Equation (4). By means of the obtained values of the observed reaction rates, a dependence on the concentration of dATP in the reaction was plotted and then calculated using Equation (5) (Figure 8c). The calculated value of *k*_pol_ was 1.3 times higher than that for WT Polβ (Table 6). It is possible that the introduction of the G290C substitution does not affect the catalytic stage. On the other hand, *K*_d,app(dATP)_ was almost 4 times higher as compared to WT Polβ. It can be concluded that the overall decrease in the polymerase activity of the G290C variant is related to effects on the stage of binding to dNTP, as well as to the weaker affinity for DNA shown above, but not to the impact of the substitution on catalysis.

**Figure 9:**
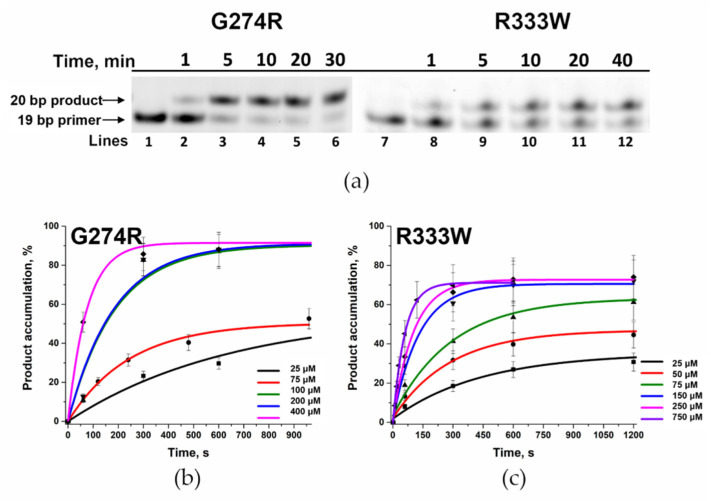
(**a**) Accumulation of the product of the gap-filling reaction performed on substrate Gap_T DNA by polymorphic variants G274R and R333W. The enzyme concentration was 0.5 μM, DNA concentration was 0.5 μM, and dATP concentration was 100 and 75 μM for G274R and R333W, respectively. Lanes 1 and 7 contain the FAM-labeled Gap_T substrate, lanes 2, 3, 4, 5, and 6 correspond to time points of 1, 5, 10, 20, or 30 min of the reaction with the Polβ G274R variant. Lanes 8, 9, 10, 11, and 12 represent time points of 1, 5, 10, 20, and 40 min of the reaction with variant R333W. The dependence of the product accumulation on time at various concentrations of dATP in the reaction is depicted in panels (**b**,**c**) for polymorphic variants G274R and R333W, respectively.

**Figure 10:**
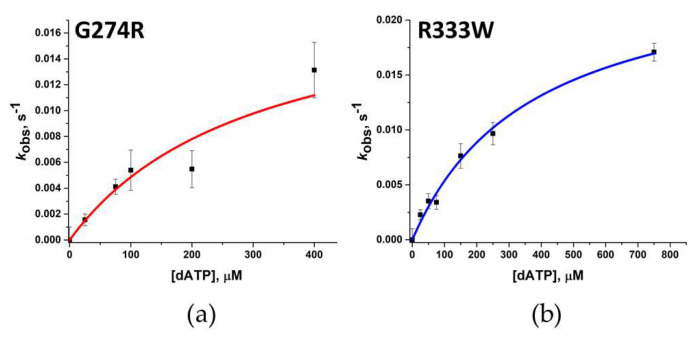
The dependence of calculated values of the observed reaction rate constants on the dATP concentration is displayed in panels (**a**,**b**) for the polymorphic variants G274R and R333W, respectively.

For Polβ variants G274R and R333W, for which it was impossible to register changes in 2-aPu fluorescence intensity, observed rate constant *k*_pol_ and *K*_d,app(dATP)_ were determined by detecting the accumulation of the polymerase reaction product with the help of PAAG electrophoresis at high concentrations of complementary dATP and a longer time scale. With an increase in these parameters, it was possible to register the accumulation of the reaction product (Figure 9a). The *k*_pol_ and *K*_d,app(dATP)_ values were determined by plotting the dependences of the observed reaction rate constant (calculated via Equation (3)) on the concentration of triphosphates in the reaction (Figure 9b,c). The resulting dependence was approximated as Equation (5) (Figure 10a,b).

For both variants G274R and R333W, an increase in *k*_pol_ and *K*_d,app(dATP)_ was observed (Table 6). Polβ variants G274R and R333W showed an approximately 20-fold weakening of dATP-binding affinity and a 50-fold decrease in polymerization efficiency; these data reflect the influence of these amino acid substitutions on all the analyzed stages of the interaction of Polβ with the substrates. For previously studied Polβ variants at position 333, a high degree of enzyme inactivation has been documented too, varying with the type of substituted amino acid residue [29].

For a more detailed consideration of the effect of substitutions G274R, G290C, and R333W on the dNTP-binding stage of the catalytic reaction, MD simulations of the complex of the enzyme with DNA and dNTP (ternary closed complex) were carried out. 

**Figure 11:**
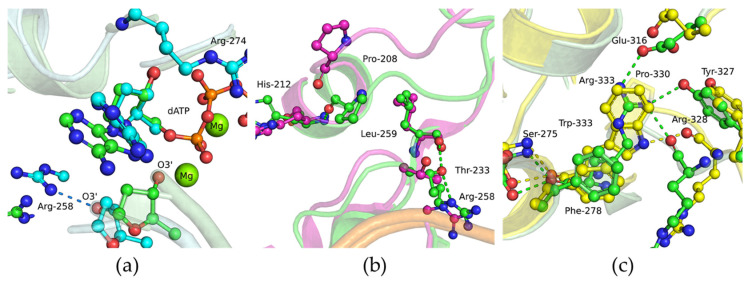
Snapshots of the micro arrangement of the protein–DNA–dNTP ternary complex involving variant G274R (**a**), G290C (**b**), or R333W (**c**) compared with WT Polβ. Salt bridges between amino acid side chains and the sugar-phosphate backbone are presented as dashed lines. WT Polβ: green, G274R: cyan, G290C: magenta, and R333W: yellow.

Replacing Gly-274 with Arg led to the possible conformations involving a change in the coordination of the 3′ oxygen atom of the primer nucleotide: this atom became coordinated with Arg-258 (Figure 11a). This conformation made it impossible to attach a new nucleotide monophosphate. Incorrect coordination of the primer nucleotide resulted in a decrease in the polymerization constant of the G274R variant by almost 50-fold, as compared to WT Polβ.

In MD simulations of the G290C variant, the location and positioning of the Cys-290 residue remained the same as in WT Polβ. Nevertheless, the alterations affected the flexible loop (aa 203–209) (Figure 11b). In WT Polβ, Pro-208 is predominantly located at the beginning of the α-helix and can form the N(Pro-208)–O(His-212) hydrogen bond. In the polymorphic variant, Pro-208 is mostly turned toward the loop. Besides, a redistribution of Thr-233 contacts was noted. In WT Polβ, Thr-233 interacts with a DNA phosphate group, the backbone oxygen atom of Leu-268, and the side chain of Arg-258. In the G290C polymorphic variant, Thr-233 is turned predominantly toward the solution or DNA. 

The MD simulations of the R333W variant’s micro positioning in the ternary closed complex did not reveal appreciable differences from WT Polβ (Figure 11c). Nonetheless, it has been previously shown that replacing Arg-333 with other amino acid residues causes a loss of contacts necessary for the normal functioning of Polβ [29]. According to MD simulations of the R333E mutant, Glu-333 moves away from the site that was previously occupied by Arg-333 in the WT structure. The relocation of Glu-333 leads to a considerable rearrangement of the main chain and side chain positioning of residues Tyr-327, Arg-328, and Glu-329. It was shown that the intact triadic interaction between Arg-182, Glu-316, and Arg-333 is required for proper polymerase function.

## 4. Conclusions

DNA polymerase β is an enzyme that take part in many processes in the cell that are aimed at preserving genetic information. Today, many mutants of the enzyme are known to occur in various types of tumors. Some of these variants are caused by SNP-induced amino acid substitutions. Several of these amino acid substitutions in Polβ may not have a considerable effect on the functioning of the enzyme; however, many Polβ mutations are known that lead to a decrease in polymerase activity and in the fidelity of the addition of a complementary nucleotide. In this work, Polβ polymorphic variants G274R, G290C, and R333W were characterized, which feature amino acid substitutions located in the dNTP selection subdomain (responsible for the correct addition of a complementary dNMP). By CD spectroscopy, it was shown that these amino acid substitutions do not induce considerable alterations in the protein globule, and measurement of the melting temperature point of the enzymes did not reveal valuable differences from WT Polβ. Using molecular dynamics methods, it was shown that substitutions G274R and R333W lead to a greater extent to the redistribution of local contacts during the interaction of the enzyme with DNA. In contrast, in G290C, amino acid substitution did not lead to a major change in local contacts with DNA. Experimental testing of the ability of the Polβ variants G274R, G290C, and R333W to bind DNA containing a 1-nt gap detected a slight decrease in DNA-binding constants. Moreover, the *K*_d_ values for the G290C and R333W variants were higher than for the G274R variant. However, the *K*_d_ values for all three variants were higher than for the WT Polβ, which is in agreement with the MD analysis of the redistribution of local contacts.

Kinetic analysis of enzymatic properties of the Polβ variants uncovered a greater degree of inactivation in variants G274R and R333W compared to WT Polβ. MD analysis revealed no possible conformations in which the G274R variant can support the transferase reaction. The data obtained for the R333W variant suggested the importance of Arg-333 for Polβ functioning. Variant G290C was active but turned out to be a slower polymerase than WT Polβ, owing to a weaker affinity for DNA, as well as an increase in observed binding constant *K*_d, app (dATP)_. An MD simulation revealed differences in the microenvironment of the substituted amino acid residues; these alterations may be a reason for the lowered activity of the Polβ variants under study.

In general, our examination of the enzymatic properties of the natural polymorphic variants of Polβ G274R, G290C, and R333W allows us to outline the influence of these amino acid substitutions on enzyme activity. For all variants, a decrease in polymerase activity was shown due to the redistribution of local contacts. The observed decrease in enzymatic activity may lead to a higher number of unrepaired DNA damage sites, which in turn leads to an increase in the number of mutations in the genome and thus can lead to malignant transformation of the cell.

## Figures and Tables

**Table 1:** Occurrence of amino acid substitutions in the dNTP selection subdomain of Polβ for various types of tumors.

aa Substitution	HIVE Biochemistry (https://hive.biochemistry.gwu.edu accessed on 21 February 2021)	COSMIC (https://cancer.sanger.ac.uk accessed on 21 February 2021)	cBioportal (https://www.cbioportal.org accessed on 21 February 2021)
L270P		Hepatocellular carcinoma	Hepatocellular carcinoma
G274V		Malignant melanoma	Cutaneous melanoma
I277V		Uterine endometrioid carcinoma	Uterine cancer
K280N	Lung squamous cell carcinoma	Squamous cell lung carcinoma	Lung cancer
N281S	Hepatocellular carcinoma		Blastoma
M282I	Cervix squamous cell carcinoma	Squamous cell cervical carcinoma	Cervical cancer
R283S	Breast carcinoma		
A284V		Uterine endometrioid carcinoma	Uterine cancer
A286S			Liver cancer
A286V	Colon adenocarcinoma		
E288K	Breast carcinoma	Invasive ductal carcinoma	Breast cancer
G290D			Uterine cancer
F291L	Upper aerodigestive tract squamous cell carcinoma		
Y296D	Small cell lymphocytic lymphoma		
R299C	Colon adenocarcinoma		
R299S	Upper aerodigestive tract squamous cell carcinoma	Head and neck squamous cell carcinoma	
P300L	Malignant melanoma		
G305E	Metaplastic breast carcinoma		Breast cancer
A307T	Malignant melanoma		
E309K	Transitional cell carcinoma		
P310L	Malignant melanoma		
D314Y	Acute myeloid leukemia		Hematological cancer
K317I	Malignant melanoma		
I319V	Glioma	Astrocytoma	Malignant glioma
D321N	Colon adenocarcinoma		
Y322C	Colon adenocarcinoma		Melanoma
W325L	Malignant melanoma	Cutaneous melanoma	
R328Q	Malignant melanoma		
D332N	Merkel cell carcinoma		
R333W	Prostate adenocarcinoma		Prostate cancer
R333Q	Cecum adenocarcinoma	Colon adenocarcinoma	Colorectal cancer

**Table 2:** Sequences of oligodeoxyribonucleotides (containing a gap with 2′-deoxyribose phosphate) used in the work.

Name	Sequence
Pol16	5′-TAGTCACCTCAATCCA-3′
Pol19	5′-GCCTCGCAGCGGTCCAACC-3′
Pol19_FAM	FAM 5′-GCCTCGCAGCGGTCCAACC-3′ *
Pol36_T_Ã	5′-TGGATTGAGGTGACTÃNGGTTGGACGGCTGCGAGGC-3′ *
Pol36_N:	5′-TGGATTGAGGTGACTANGGTTGGACGGCTGCGAGGC-3′ *

* where N = A/T/G/C, Ã = 2-aPu residue, and FAM = 6-fluorescein residue.

**Table 3:** Calculated contents of α-helices and calculated melting points of the Polβ variants.

	WT	G274R	G290C	R333W
α-helices, %	79 ± 16 *	48 ± 10	33 ± 7	34 ± 7
T_m_, °C	44.9 ± 0.2 **	42 ± 1	42.5 ± 0.9	41 ± 1

* from Ref. [32]; ** from Ref. [55].

**Table 4:** Calculated dissociation constants *K*_d_ (μM) of the complexes of variant G274R, G290C, and R333W with DNA.

	Gap_A	Gap_T	Gap_G	Gap_C
WT *	0.38 ± 0.02	0.33 ± 0.03	0.59 ± 0.07	0.38 ± 0.03
G274R	1.10 ± 0.09	1.4 ± 0.1	1.2 ± 0.1	1.4 ± 0.1
G290C	1.0 ± 0.1	1.8 ± 0.1	2.4 ± 0.2	1.6 ± 0.1
R333W	4.4 ± 0.2	3.6 ± 0.4	4.9 ± 0.6	3.6 ± 0.2

* from Ref. [32].

**Table 5:** Observed reaction rate constants *k*_obs_ for the incorporation of a complementary nucleotide into the tested DNA substrates containing a 1-nt gap.

	Gap_A	Gap_T	Gap_G	Gap_C
WT *	0.33 ± 0.03	0.32 ± 0.04	0.25 ± 0.02	0.34 ± 0.02
G290C	0.12 ± 0.02	0.081 ± 0.002	0.063 ± 0.003	0.10 ± 0.02

* from Ref. [32].

**Table 6:** Chemical reaction rate constants *k*_pol_ and observed dissociation constants *K*_d,app(dATP)_.

	*k*_pol_, s^−1^	*K*_d,app(dATP)_, µM
WT *	0.93 ± 0.05	16 ± 3
G274R	0.019 ± 0.008	306 ± 168
G290C	1.2 ± 0.1	62 ± 21
R333W	0.025 ± 0.002	374 ± 75

* from Ref. [32].

## Data Availability

Raw experimental data are available from O.A.K. and A.A.K. upon request. Tel. +7 (383) 363-5174, E-mail: kladova@niboch.nsc.ru (O.A.K.); sandra-k@niboch.nsc.ru (A.A.K.).

## Supplementary Materials

The following supporting information can be downloaded at: https://www.mdpi.com/article/10.3390/biom14050547/s1, Original Western Blots images.
