# Supplementary figures and images for: SNP-Associated Substitutions of Amino Acid Residues in the dNTP Selection Subdomain Decrease Polβ Polymerase Activity
Source: Biomolecules. 2024 May 2;14(5):547. doi: 10.3390/biom14050547 (PMC11117729; doi:10.3390/biom14050547)

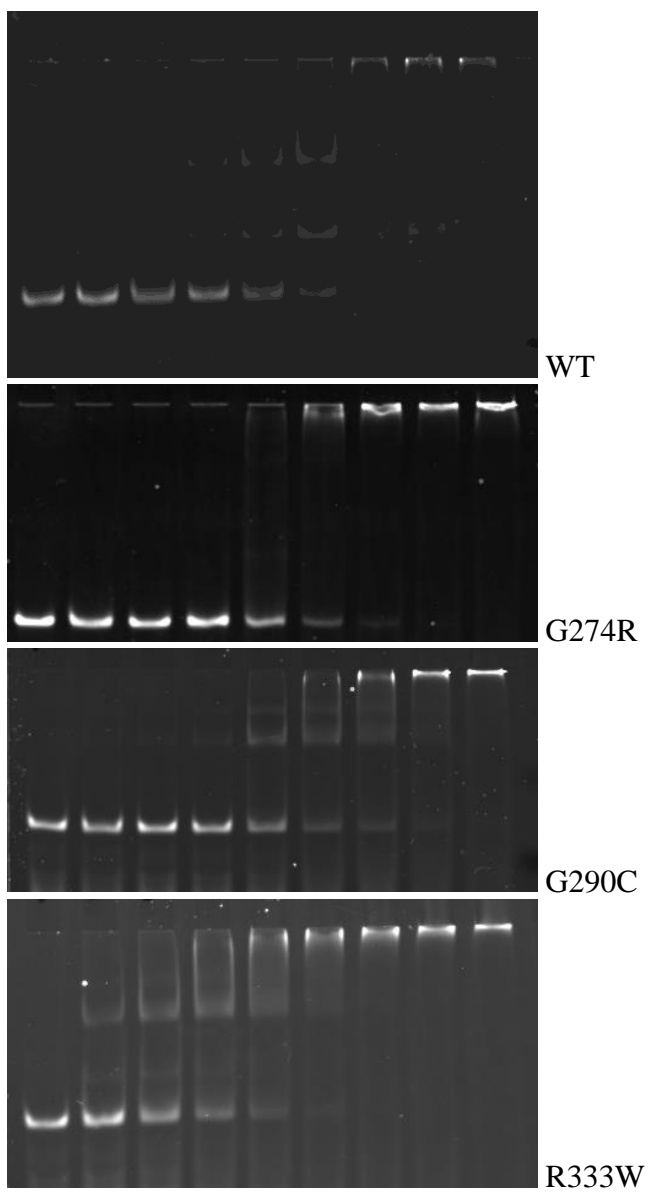

The original figures in Figure 4.

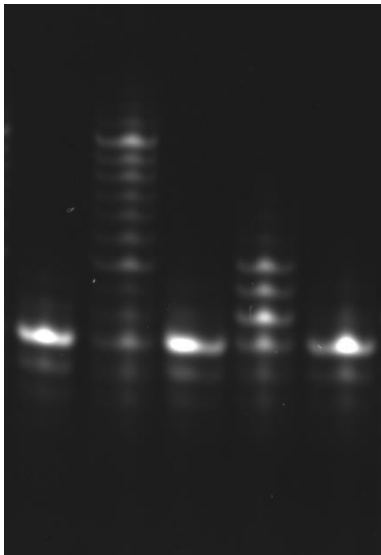

The original figures in Figure 7.

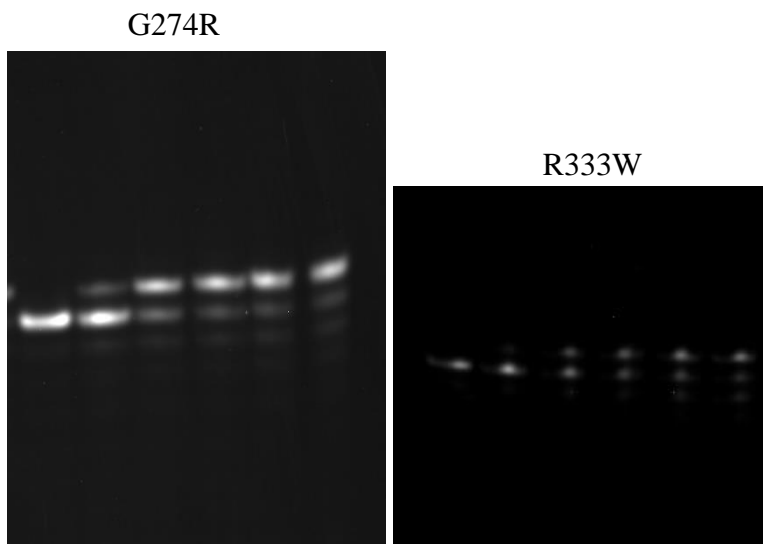

The original figures in Figure 9.

Supplement: Supplementary file 1 [file biomolecules-14-00547-s001.zip › biomolecules-2960652-supplementary.pdf]
